# Creation of an mHealth Infrastructure to Support the Development and Delivery of mHealth Interventions: Protocol for Demonstration Projects Addressing Smoking Cessation in Cancer Care

**DOI:** 10.2196/92288

**Published:** 2026-05-29

**Authors:** Lindsey S Sparrock, Jennifer I Vidrine, Christine E Vinci, Issam M El Naqa, Steven K Sutton, Ramzi G Salloum, Jesse Dallery, Tracy E Crane, Frank J Penedo, Samuel J Brockway, Sarah R Jones, Charles E Hoogland, Richard R Reich, Guillermo Gonzalez-Calderon, Vani N Simmons, Damon J Vidrine

**Affiliations:** 1 Department of Health Outcomes & Behavior Moffitt Cancer Center Tampa, FL United States; 2 Department of Psychology College of Arts and Sciences University of South Florida Tampa, FL United States; 3 Department of Oncologic Sciences Morsani College of Medicine University of South Florida Tampa, FL United States; 4 Department of Machine Learning Moffitt Cancer Center Tampa, FL United States; 5 Department of Radiation Oncology Moffitt Cancer Center Tampa, FL United States; 6 Department of Gastrointestinal Oncology Moffitt Cancer Center Tampa, FL United States; 7 Department of Biostatistics and Bioinformatics Moffitt Cancer Center Tampa, FL United States; 8 Department of Health Outcomes and Biomedical Informatics College of Medicine University of Florida Gainesville, FL United States; 9 Health Cancer Center University of Florida Gainesville, FL United States; 10 Department of Psychology University of Florida Gainesville, FL United States; 11 Department of Medicine Miller School of Medicine University of Miami Miami, FL United States; 12 Department of Psychology University of Miami Coral Gables, FL United States

**Keywords:** mHealth, smoking, randomized controlled trial, feasibility, tobacco cessation, cancer

## Abstract

**Background:**

Cancer remains a leading cause of morbidity worldwide. To reduce this burden, scalable, effective approaches are needed to address modifiable risk factors for cancer and support behavioral self-management. With smartphone ownership now nearly ubiquitous, mobile health (mHealth) interventions offer a powerful means to extend the reach, accessibility, and sustainability of evidence-based treatments for a variety of modifiable risk factors (eg, excessive alcohol use, physical inactivity, poor diet, and smoking). Moreover, the flexibility of mHealth platforms enables efficient delivery of novel interventions, supports innovative study designs, and facilitates real-time data collection to advance public health research.

**Objective:**

Despite the great potential of mHealth interventions, developing high-quality mHealth tools is complex, time-consuming, and resource-intensive. To address these challenges, we are developing a coordinated, accessible, research-grade infrastructure for mHealth app development, testing, and dissemination.

**Methods:**

The mHealth Florida infrastructure (mFLi) will provide a comprehensive, low-code software platform that enables researchers to build apps compatible with major mobile operating systems, namely, Apple iOS and Google Android. Through a modular interface, users will select from a menu of prebuilt features to tailor functionality to specific study needs. The platform will include 3 integrated environments (development, testing, and production), allowing researchers to prototype, evaluate, and deploy mHealth interventions. This infrastructure will be developed and maintained by a multidisciplinary team, ensuring that the platform is technically robust and usable and adheres to institutional and regulatory standards. To demonstrate the platform’s functionality, utility, and adaptability, a multisite study comprising three initial projects focused on smoking cessation among patients with cancer is being conducted: (1) participant screening and enrollment, (2) randomization and treatment delivery, and (3) data processing using machine learning methods with on-device and cloud-based approaches.

**Results:**

This study was funded in May 2023, and ethics approval was obtained from all involved sites’ institutional review boards between February 2024 and October 2025. Recruitment began in March 2025 and enrollment is ongoing. As of January 2026, 41% (37/90) of the target sample have been enrolled and 21% (19/90) have completed their 6-month assessment. Data collection will be completed once the final participant completes their 6-month assessment (expected May 2027), with analyses commencing thereafter. Study findings are anticipated to be published in a peer-reviewed journal in 2027.

**Conclusions:**

Collectively, these projects will illustrate how mFLi can streamline app development, facilitate rapid translation of research into practice, and reduce barriers for researchers and developers. Ultimately, mFLi is designed to accelerate innovation in mHealth research, enhance access to behavioral interventions, and improve health outcomes among diverse populations.

**Trial Registration:**

ClinicalTrials.gov NCT06909357; https://clinicaltrials.gov/study/NCT06909357

**International Registered Report Identifier (IRRID):**

PRR1-10.2196/92288

## Introduction

### Background

Cancer is a leading cause of mortality worldwide [1]. Unhealthy behaviors, such as alcohol consumption, poor diet, physical inactivity, and smoking, are well-established, modifiable risk factors for cancer [2,3], contributing to its onset, progression, and recurrence. A potential strategy to reduce the growing global cancer burden is implementing evidence-based behavioral interventions to promote the adoption and maintenance of healthy behaviors. To be impactful, these interventions must demonstrate not only effectiveness but also broad reach, acceptability, and sustainability. Despite extensive evidence supporting the acceptability and efficacy [4-6] of traditional behavioral interventions (eg, quitlines), their impact remains limited, partially due to challenges with funding, widespread reach [7-9], and sustainability [10]. That is, barriers such as cost, accessibility, scalability, lack of awareness, and lack of personalization often restrict the reach and long-term impact of behavioral interventions for these modifiable risk factors [11,12], particularly in resource-constrained settings and among socioeconomically disadvantaged populations.

### Mobile Health

Mobile health (mHealth) technologies offer a promising solution to these challenges. With smartphone ownership exceeding 90% among adults in the United States, including high penetration among racial and ethnic minority groups, individuals with low socioeconomic status, and rural populations [13], mHealth interventions have the potential to overcome many traditional barriers to participation by providing low-cost, scalable, personalized, and accessible health support. Evidence suggests that mHealth approaches can be effective for a variety of health behaviors, including smoking cessation [14], alcohol consumption [15], physical activity [16], dietary changes [17], and chronic disease management [18]. In some cases, mHealth interventions have demonstrated equal or greater effectiveness compared with traditional treatments [19]. Furthermore, previous findings indicate that mHealth interventions may be more cost-effective than traditional interventions, suggesting potential benefits in both costs and health outcomes [20,21]. Overall, the advantages of mHealth (eg, real-time monitoring, low-cost, personalization, and sustained reach) underscore its potential to transform behavioral health interventions.

The consumer market for mHealth apps is rapidly expanding, with thousands of health-related apps currently available across commercial platforms [22]. While several evidence-based and theoretically informed mHealth apps exist [23], most commercially available apps are not evidence-based, lack grounding in established behavioral theories, lack involvement from academic or public health entities, and have not undergone rigorous evaluation [24-28]. The development of mHealth apps is a complex, resource-intensive process. Common challenges include the need to meet stringent data security and privacy requirements, addressing the technical complexity of building research-grade apps that comply with both clinical and regulatory standards, and managing the frequent disconnect between stakeholders (eg, academic researchers) and commercial developers [29-31]. Furthermore, app development can be costly, with reported average costs up to US $425,000 [32,33] depending on various factors, such as the app’s type (eg, informational, diagnostic, disease management, and fitness tracking [34]), features (eg, scheduling, reminders, alarms, and gamification), regulatory and compliance requirements, and target platform. These costs present a significant barrier to many researchers, particularly given that the overall biomedical research landscape has faced considerable challenges due to recent policy changes that have reduced grant success rates and total funding [35,36]. Moreover, development timelines are often misaligned with traditional research funding cycles [30], which can make dissemination and sustainability challenging as ongoing updates, maintenance, and long-term funding are required to keep apps functional.

### Our Study

Researchers often face considerable challenges when developing evidence-based mHealth behavioral interventions. To address these barriers, we are creating the mHealth Florida infrastructure (mFLi), a statewide resource designed to accelerate the creation, testing, and deployment of research-grade mHealth apps. The overarching goal of this infrastructure is to support behavioral research and address participant needs by providing a coordinated, accessible, and secure environment for mHealth app development. Specifically, the infrastructure is designed to (1) support research recruitment and screening and (2) ensure seamless integration of app-based functions, including randomization, referral, personalized intervention delivery, data collection, and real-time monitoring.

In addition to describing the design and components of mFLi, this paper presents 3 demonstration projects that showcase and assess its functionality in supporting mHealth research. Specifically, these demonstration projects will serve as proof-of-concept applications highlighting the infrastructure’s capacity to support patient screening and recruitment (demonstration 1), randomization and intervention delivery (demonstration 2), and data processing via machine learning (demonstration 3). The proposed demonstration projects serve two key purposes: (1) to provide critical information regarding the capabilities and functionality of the infrastructure and (2) to generate valuable preliminary data to assess mFLi’s suitability for future efficacy trials testing mHealth interventions.

## Methods

### Ethical Considerations

This study was reviewed and approved by the institutional review boards at the University of Miami (IRB#20250017), the University of Florida (IRB#202400024), and Advarra (H Lee Moffitt Cancer Center; IRB#00000971). The study is registered on ClinicalTrials.gov (NCT06909357) and was reviewed and funded by the Florida Department of Health Biomedical Research Program 22-23 James and Esther King award under award number 23K01 (Multimedia Appendix 1). All study procedures will be carried out in accordance with relevant guidelines and regulations. Electronic informed consent will be obtained from all participants included in the study prior to enrollment, and participants will be informed of their right to withdraw from the study at any time without any consequences. To ensure privacy and confidentiality, participant data will be anonymized and accessible only to authorized team members. Participants will receive compensation for completing assessments at baseline and at the 1-, 3-, and 6-month follow-ups (4 assessments × US $30 = US $120).

### Phase 1: mHealth Resource Development

#### Purpose

**Figure 1:**
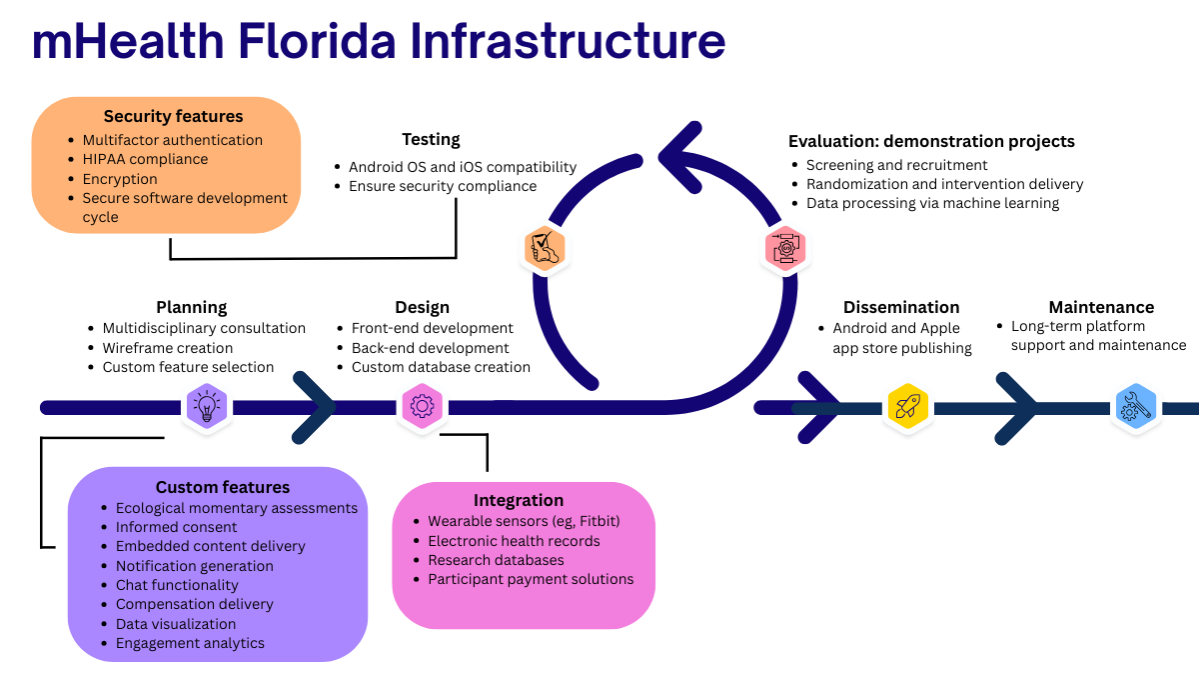
Illustration of the mobile health (mHealth) Florida infrastructure development process. Using a multidisciplinary approach, researchers will be able to plan, design, test, disseminate, and maintain mHealth tools. HIPAA: Health Insurance Portability and Accountability Act.

The mFLi platform will enable researchers to design, test, and disseminate mHealth tools (ie, web-based and native mobile apps) that primarily address behavior change and will be compatible with Apple iOS and Google Android operating systems (Figure 1). The platform will use a low-code interface, allowing users to select from a menu of modular features to meet the needs of specific projects. Available functionalities will include customizable data collection modules, complex rules engines, data monitoring dashboards, engagement analytics, and the ability to integrate with external databases and multiple sources of health-related data. To accommodate different stages of research and development, mFLi will provide three environments: (1) development, (2) testing, and (3) production. Researchers will be able to independently design, test, and publish apps, with the option to request support from a mobile app developer, a user experience (UX) and user interface (UI) designer, or both.

#### Planning, Design, and Testing

The development and maintenance of this infrastructure will require a multidisciplinary team with complementary expertise. A dedicated external software developer will lead the technical development of the platform and support the UX and UI designer and program manager. The UX and UI designer will ensure user-friendly design and participant-facing interfaces, while the program manager will coordinate effective communication and workflows between research and development teams across all stages of planning, development, and deployment.

Additionally, the Biostatistics and Bioinformatics Shared Resource Core at the Moffitt Cancer Center will co-lead the development of the platform and provide long-term platform support, including oversight of internal system security requirements, maintaining institutional compliance, and completing postdevelopment modifications as needed. Internal ownership, management, and maintenance at the Moffitt Cancer Center will enhance the platform’s feasibility, given that many external development companies incur high ongoing maintenance costs, which pose a significant financial barrier. Furthermore, oversight and guidance will be provided by 2 advisory bodies: (1) a Scientific Advisory Board, comprising researchers from institutions across Florida, which will provide input on platform design and ensure mFLi meets the diverse needs of the scientific community; and (2) a Community Advisory Board, comprising members of the Tampa Bay Community Network, which will provide iterative feedback throughout the development process to ensure that the platform supports the creation of apps appropriate for diverse populations, including underserved groups. This team-based approach will ensure that the platform is technically sound, researcher- and research-friendly, meets the needs of the community, and is compliant with institutional and regulatory requirements.

### Phase 2: Evaluating mFLi via Demonstration Projects

#### Overview

To help evaluate the mFLi platform, a pilot 2-group randomized controlled trial (RCT) comparing the feasibility of 2 smoking cessation treatments (mHealth intervention vs traditional quitline intervention) among individuals diagnosed with cancer will be conducted to demonstrate the infrastructure’s utility. The 3 phases of this demonstration project are as follows.

#### Demonstration 1: Recruitment via Ask-Advise-Connect

Participant recruitment is planned to span from March 2025 to February 2026 and will include individuals receiving cancer treatment from one of the following three Florida Academic Cancer Center Alliance institutions: (1) H Lee Moffitt Cancer Center, (2) University of Florida Health Cancer Center, and (3) University of Miami Sylvester Comprehensive Cancer Center.

Adopting a systems-level approach, we will use a digital tool developed in mFLi and modeled after the “Ask-Advise-Connect” (AAC) approach to assess smoking status and facilitate treatment enrollment. The AAC approach has been successfully implemented in primary care settings and has demonstrated increased cessation treatment enrollment probability relative to older approaches, such as “Ask-Advise-Refer” [37-40]. The Ask-Advise-Refer approach involves training medical staff to “Ask” all patients about their smoking status consistently and systematically, “Advise” all patients who smoke to quit, and “Refer” patients to quitline services. However, rather than referring patients, AAC involves training staff to “Connect” patients who agree to be contacted to either quitline services or a fully automated, digitally delivered smoking cessation intervention. Specifically, staff will either manually complete a referral connecting the patient with the Florida Quitline or generate an encrypted link sent to the patient’s email via Research Electronic Data Capture (REDCap; Vanderbilt University) containing download links for their operating system–specific app store and unique account information for the patient that unlocks access to the full app. Staff will be available to provide further assistance with downloading and activating the app.

All patients will be identified either through in-person or remote approaches. Patients presenting at one of the clinics will be approached in person and provided a tablet by staff members to complete the AAC prescreening survey. Furthermore, patients who have received care from one of the clinics in the past 120 days will be identified via Moffitt Cancer Center’s Collaborative Data Services Core, which integrates patient information from multiple clinic sources, including the electronic health record (EHR). Records will be systematically reviewed to ensure that patients have not previously completed the AAC prescreening survey and to eliminate duplicate entries. Contact information (eg, phone numbers and email addresses) will then be entered into a REDCap survey invitation list. Patients who have not yet completed the prescreening survey will be approached via email or SMS text message to assess initial eligibility for a smoking cessation study.

To determine initial eligibility, a brief 5-item (approximately 1-min) AAC prescreening survey assessing smoking and other health behaviors (eg, diet, physical activity, and medication use) will be administered to all patients via mFLi’s REDCap, with responses from patients approached in person uploaded automatically to a custom dashboard. On the basis of a conservative estimate of 10% smoking prevalence among patients with cancer, and recent estimates of smoking cessation treatment enrollment among patients with cancer averaging around 30%, we estimate that 3000 patients (approximately 90% of patients approached) will need to complete the AAC prescreener. We estimate that this will lead to the identification of approximately 300 current smokers and the enrollment of 90 current smokers (approximately 30% of eligible patients) in demonstration project 2. Consistent with the National Cancer Institute’s definition of current smoking for patients with cancer [41], patients who self-report smoking in the past 30 days will automatically receive a brief, digitally delivered educational video message advising them to quit (eg, “Quitting smoking is the most important thing you can do to improve your response to treatment and your overall health”). Following the brief advice to quit, participants will be able to opt out of follow-up contact from the study team regarding available cessation resources.

##### Primary Outcome Measures and Analytic Approach

In demonstration project 1, we will use descriptive statistics to evaluate feasibility metrics related to screening, recruitment, and enrollment processes, including the total number of patients screened for tobacco use, the proportion of patients who complete the smoking question on the screener, the proportion of current smokers provided automated advice to quit, and the proportion of current smokers who agree to enroll in a smoking cessation treatment program. mFLi’s recruitment and initial screening capability will be deemed feasible via the demonstration project if at least 90% of patients approached complete the prescreener, the proportion of patients identified as current smokers meets or exceeds the historical (prior 2 years) self-reported proportions recorded in the EHRs at each of the 3 partnering institutions, and at least 30% of eligible patients agree to enroll in the smoking cessation RCT (demonstration project 2).

#### Demonstration 2: Screening, Randomization, Intervention Delivery, and Assessments

##### Screening and Consent

Following receipt of smoking status information in demonstration project 1, potentially eligible patients who agree to enroll in treatment and who do not opt out of receiving information will then be directed to complete the main screener. Specifically, those who are recruited in person will have their information in the dashboard automatically transferred to mFLi’s REDCap and will be automatically sent a brief message and link to the main screener via SMS text message or email. Those recruited remotely will be seamlessly transitioned from the AAC prescreener to the main screener. Those who do not respond to the automated link will be contacted by research staff and asked to complete the screener. Study inclusion criteria are (1) aged 18 years or older, (2) self-report of smoking within the last 30 days and a history of at least 100 lifetime cigarettes, (3) ownership of a working smartphone, and (4) ability to speak English. Exclusion criteria are (1) failure to complete study enrollment within 14 days of completing the screener, and (2) being pregnant or breastfeeding. Patients meeting eligibility criteria will be invited to participate and provided with a detailed description of the study. All individuals who agree to enroll will complete a Health Insurance Portability and Accountability Act (HIPAA)–compliant electronic informed consent process.

##### Baseline Assessment

Participants who consent to participate will be instructed to complete baseline measures, including demographics, subjective social status [42], health literacy [43,44], quit motivation [45], smoking cessation self-efficacy [46], perceived stress [47], the Patient Health Questionnaire depression scale [48], Cancer Patient Tobacco Use Questionnaire [49], and nicotine dependence [50].

##### Randomization and Blinding

After completing the baseline measures, participants (N=90) will be randomized 1:1 within mFLi to either a standard quitline treatment (QT; n=45) or a prototype version of a fully automated, digitally delivered smoking cessation treatment, Digital Motivation and Problem Solving (D-MAPS; n=45), using simple randomization sequences generated by the study statistician. Due to the nature of the protocol study and trial design, blinding of participants and the study statistician to treatment assignment was not feasible or necessary.

##### Intervention Delivery

Participants randomized to the QT group will receive a 10-week supply of combination nicotine replacement therapy (patches and lozenges) and have their information sent to Tobacco Free Florida (a HIPAA-covered entity). Quitline staff will then proactively contact these individuals and present treatment options (eg, phone counseling, group counseling, and web-based services). Quitline counseling approaches are recommended in the Treating Tobacco Use and Dependence Clinical Practice Guideline as an evidence-based treatment option [51].

The D-MAPS treatment approach is based on the previously developed human-delivered Motivation and Problem Solving approach [52-54]. Participants randomized to the D-MAPS group will receive 10 weeks of combination nicotine replacement therapy and will be provided (via email and/or SMS text message) a link to download the app. The app-based intervention will comprise 3 distinct and personalized sessions (session 1—“Getting started,” session 2—“Continuing your journey,” and session 3—“Maintaining your progress”). In each session, users will complete brief assessments and, regardless of readiness to quit smoking, be given the option to create a wellness plan (eg, stress management, alcohol use, nutrition, physical activity, sleep, and finances). The intervention is designed for participants to repeat sessions, and participants’ responses and selections in each session will guide the delivery of dynamically tailored content (animated videos, audio, and text) to ensure a personalized UX responsive to individual needs and real-time changes in motivation or behavior. This intervention will integrate principles drawn from motivational interviewing, social cognitive theory, and cognitive behavioral therapy [55-57]. Participants will be proactively sent prompts via push notifications based on their scheduling preferences, and they can also access smoking cessation and wellness plan content on demand. Participants will be able to use D-MAPS over a 6-month intervention period.

##### Wearable Sensor

Additionally, participants in both conditions will be provided with commercially available wearable wrist sensors (eg, Fitbit devices; Google LLC) and instructions for setup. These devices automatically and unobtrusively collect a range of health-related data, including physical activity data (accelerometer, gyroscope, and active minutes), as well as health and wellness data (heart rate, blood oxygen saturation, and skin temperature variation). Participants will be instructed to wear the sensors daily throughout the 6-month study. Specifically, participants will be asked to wear the sensors for their waking hours, or up to 16 hours per day, for the duration of the study. They will only be advised to remove the device while charging, during any extended water exposure (eg, showering or swimming), and in the event of irritation or skin reaction. At the completion of the study, participants will be permitted to keep the provided device. Fitbit data will be integrated with all app-based and assessment data.

##### Ecological Momentary Assessment

Participants in both treatment groups will be prompted to complete ecological momentary assessments (EMAs) once per day during the initial 8 weeks, and once per week for the remainder of the study (4 months) via push notification. The method by which participants will complete EMAs will differ by treatment group, with QT participants completing EMAs online via REDCap and D-MAPS participants completing EMAs via the app. Using EMAs will allow us to examine the dynamic impacts of multiple psychological, social, and environmental factors during a quit attempt. Specifically, participants will be asked questions relating to their current smoking status, perceived stress level, smoking self-efficacy, motivation to quit smoking, and nicotine replacement therapy adherence [58].

##### Primary Outcome Measures

The primary outcome measures for demonstration project 2 will be the feasibility of the D-MAPS app, assessed via usability and acceptability. Specifically, usability will be assessed with the 10-item System Usability Scale [59], as well as through a single item assessing the percentage of participants who rate the app as “easy to use.” It will be deemed usable if at least 80% of participants score >70 (range 0-100) on the System Usability Scale and at least 80% of participants respond “yes” (dichotomous yes or no) to the app being “easy to use.” The acceptability of and satisfaction with the D-MAPS app will be assessed via the Client Satisfaction Questionnaire [60]. It will be deemed acceptable and satisfactory if (1) at least 80% of participants score >3 (range 1-4) on the Client Satisfaction Questionnaire, (2) at least 80% of participants report being mostly or very satisfied with the intervention, (3) at least 80% of participants respond “yes” to recommending the intervention to a friend to quit smoking, and (4) at least 80% of participants rate the quality of the intervention as good or excellent. Finally, various app engagement metrics will be measured (eg, video playback duration and frequency of app access).

##### Analytic Approach

This study is not designed or powered to test significant differences between our 2 conditions in demonstration project 2. However, we will describe cessation outcomes in both treatment conditions using 7-day point prevalence abstinence at each follow-up and calculate CIs. These data, combined with feasibility metrics, will be useful when designing a future, fully powered trial.

#### Demonstration 3: Data Collection and Processing via Machine Learning

##### Overview

Machine learning and deep learning, subfields of artificial intelligence, offer promising tools for identifying complex patterns in health-related data via computer algorithms in multiple domains, including cancer [61]. These methods are particularly well suited for analyzing data from diverse sources (eg, self-report, clinical assessments, wearable devices, and GPS) to generate predictive models of behavior. Despite this potential, machine and deep learning remain underutilized in mHealth interventions [62]. Therefore, the primary goal of demonstration project 3 is to evaluate the capacity of the mFLi platform to support the application of machine and deep learning methods for predicting key behavioral health outcomes (eg, smoking cessation and relapse). By integrating and analyzing data from the participants’ EHRs (eg, on diagnosis and treatment) and data collected from the 90 patients enrolled in demonstration projects 1 and 2 (split randomly; 70% of participants assigned to the training phase and 30% assigned to the validation and testing phases), this project aims to assess the feasibility of applying advanced analytic techniques to mHealth-collected data, thereby highlighting mFLi’s potential to shift traditional paradigms in behavioral research and support next-generation personalized interventions.

##### Data Processing: Machine and Deep Learning

The application of machine and deep learning algorithms to mHealth can be enabled via two approaches: (1) cloud-based client server and (2) on-device app.

In cloud-based client server setup, the machine learning and deep learning algorithms are hosted in the cloud (server), with access to powerful computing resources (graphics processing units), and the data are sent from the mobile device (client) over the internet to the cloud. The main advantage in this case is that conventional and complex machine learning algorithms can be directly used. In such a scenario, the analysis can begin with conventional ensemble machine learning techniques, including random forests, which are robust to heterogeneous data types and have demonstrated strong performance in prior related studies [63-66]. The importance of different factors will be ranked using methods based on the Gini index (eg, permutation testing), and associated statistical metrics will be used to assess their significance. To address limitations inherent in traditional machine learning approaches, we will also explore deep learning models, which integrate feature extraction directly into the predictive modeling process [67]. These models are particularly well suited for large, high-dimensional datasets. To enhance model interpretability, we will use techniques such as Shapley Additive Explanations values and interpretability maps, allowing us to rank order key predictors contributing to model outcomes [68-70]. If sample size limits the performance of deep learning models, we will use hybrid models that combine both conventional machine learning and deep learning techniques [71]. The main disadvantages in this case are latency and privacy concerns.

In on-device app setup, the machine and deep learning algorithms can run directly on the mobile device. Typically, the algorithms are developed offline as described earlier (training mode), then deployed on the device (inference mode). The inference mode is less computationally demanding and can benefit from existing frameworks such as Core ML (Apple Inc) on Apple iOS and TensorFlow Lite (Google LLC) on Android systems. This approach provides advantages such as real-time performance, offline functionality, and improved privacy by maintaining the data secured on the device. This approach will be favored in our design.

##### Handling Missing Data

We will make every effort to keep missing data at a minimum. We will compare the characteristics of responders and participants who dropped out of the study. Additionally, several imputation strategies (eg, last value carried forward, regression approaches, and missing equals smoking) will be used as sensitivity tests.

## Results

This study was funded by the Florida Department of Health (23K01) in May 2023 and approved by the institutional review boards at the University of Miami (approved April 2025), University of Florida (approved October 2025), and Moffitt Cancer Center (approved February 2024). Recruitment for the study began in March 2025 and is ongoing, with enrollment expected to be finalized in November 2026. As of January 2026, 41% (37/90) of the target sample have consented and been enrolled into the study. Participants are followed for 6-months after baseline; as of January 2026, 21% (19/90) have completed the 6-month follow-up assessment. Data collection is expected to be completed once the final participant completes the 6-month assessment (expected May 2027). Following data collection, data will be analyzed and findings will be disseminated.

## Discussion

### Expected Outcomes and Potential Impact

The purpose of this study is to develop mFLi and to evaluate the feasibility of the platform via 3 demonstration projects. The results from this study will address existing challenges related to developing evidence-based mHealth treatments (eg, complicated, time-intensive, and resource-intensive) by supporting, streamlining, and reducing the costs of developing and disseminating digital interventions. Specifically, results will be critical for informing future mFLi infrastructure changes, including, but not limited to, the following areas: randomization, e-consent, EMA, automated delivery of financial compensation, notifications, and delivery of content. We also anticipate that results from this demonstration project will provide support for a subsequent RCT and will inform the need for additional modifications to the app prior to efficacy testing. Thus, mFLi may enhance the efficacy of digital health interventions, health outcomes, and health care delivery.

### Dissemination Plan

The findings from this study will be disseminated through peer-reviewed publications, conference presentations, and meetings with relevant stakeholders. We will leverage existing relationships with our advisory boards (Scientific Advisory Board and Community Advisory Board) and funders to identify key stakeholders who may benefit from or contribute to the dissemination of findings from this and subsequent studies. These partnerships will also inform ongoing refinement and development of mFLi. In collaboration with stakeholders, we will also explore opportunities for future funding and resources to support broader implementation and dissemination efforts.

### Clinical Relevance and Potential Strengths

The infrastructure has several unique strengths, including (1) reducing the burden on researchers desiring to develop mHealth interventions and on diverse individuals and populations seeking personalized, cost-effective, and evidence-based treatment; (2) enabling multi-device integration (ie, wearable devices) through a flexible architecture that allows for the collection, integration, and analysis of multiple data sources; (3) embedding robust security, regulatory, and compliance guardrails for users across internal and external institutions; (4) featuring flexible design capabilities allowing future apps to leverage existing features or develop new functionality as technology and needs evolve; and (5) involving multiple partners including 3 organizations with different structures.

### Potential Limitations

The proposed infrastructure also has anticipated limitations that should be mentioned, including requiring all users to own a smartphone with an operating system compatible with the project app (Apple iOS or Google Android operating systems); however, smartphone ownership is now nearly ubiquitous (91% of US adults own a smartphone [13]) and iOS and Android combined encompass nearly all of the US mobile operating system market (iOS 58% and Android 42% [72]); requiring at least a modest degree of digital literacy; however, most US adults have basic computer skills and can be considered digitally literate (84%) [73] and evidence suggests that individuals with lower digital literacy are interested in and receptive to digital cessation treatments [74]; and requiring users to speak English; however, there are plans to include additional languages. Therefore, we do not anticipate these potential limitations will affect the feasibility or utility of this infrastructure.

### Conclusions

In conclusion, consumers need evidence-based, sustainable, and tailored mHealth interventions, while researchers need feasible, cost-effective, and streamlined infrastructure to support the development of mHealth apps. The mFLi platform has enormous potential to advance public health efforts, as its demonstrated feasibility (via the 3 demonstration projects) would support its use to facilitate the creation, testing, and implementation of a wide variety of mHealth behavioral interventions.

## Appendix

Multimedia Appendix 1 Peer review report by Florida Department of Health Biomedical Research Program.

## Abbreviations

AAC: Ask-Advise-Connect
D-MAPS: Digital Motivation and Problem Solving
EHR: electronic health record
EMA: ecological momentary assessment
HIPAA: Health Insurance Portability and Accountability Act
mFLi: mHealth Florida infrastructure
mHealth: mobile health
QT: quitline treatment
RCT: randomized controlled trial
REDCap: Research Electronic Data Capture
UI: user interface
UX: user experience
