# Supplementary material for: Creation of an mHealth Infrastructure to Support the Development and Delivery of mHealth Interventions: Protocol for Demonstration Projects Addressing Smoking Cessation in Cancer Care
Source: JMIR Res Protoc. 2026 May 29;15:e92288. doi: 10.2196/92288 (PMC13263659; doi:10.2196/92288)
Supplement: Multimedia Appendix 1 [file resprot_v15i1e92288_app1.pdf]

Review: Florida Department of Health Biomedical Research Program 22-23

Proposal Number: 20006

Proposal Title: Creation of an Infrastructure to Support Delivery of mHealth Interventions for Cancer Patients Throughout Florida

Principal Investigator Name: Vidrine, Damon

Panel: Peer Review – James and Esther King

Significance

Significance - Strengths:

Reviewer 1
This project proposes to create a research infrastructure to improve the efficiency of mHealth interventions, especially tobacco cessation treatment to cancer patients, in Florida. The proposed mHealth research infrastructure, if successful, has the potential to advance science and technology and improve clinical practice in the tobacco cessation treatment of cancer patients in Florida through developing the concept, methods and technological capability (prototype deployment) of mHealth by integrating advanced mobile and internet computing and machine learning technology.

Reviewer 2
mHealth offers many opportunities to improve patient care. Development of apps can be time consuming. Platforms such as mFLi can facilitate quick app creation which will increase use of mHealth for studying and deploying interventions.

Reviewer 3
---The access to a customizable app for individual research projects will be a substantial asset to researchers, and it will notably provide a 'edge' for competitive federal grant funding.
Broad potential for use in research as a data collection tool and/or treatment delivery method.
Broad potential applications beyond tobacco cessation to other health interventions in the future.

Significance:

Weaknesses:

Reviewer 1
The proposal can benefit from more elaboration or a couple of concrete scenarios to illustrate how AI-enhanced mHealth will help enhance the quality of current data analysis done in the context of improve clinical practice of the tobacco cessation treatment to cancer patients in Florida.

Reviewer 2
The project may achieve its Aims but still not succeed in having much impact on the field if mFLi is not used. The funding support from Moffitt partially alleviates this concern.

Reviewer 3
Minor - The applicants suggest that a device might reduce stigma in smoking status reporting compared to in-person assessment by the care team. Additional data to support this would be welcome – do more people endorse smoking, do more people engage with treatment when delivered in this method?

Investigator(s):

Strengths:

Reviewer 1
The team is strong and has extensive expertise in smoking research with cancer patients, mHealth smoking cessation interventions to special populations, including underrepresented groups, and machine learning in oncology. The PI and his collaborators are well established experts with complementary and integrated expertise in tobacco related cancer treatment and smoking cessation interventions using mobile health technologies.

Reviewer 2
The assembled team has experience in the relevant areas of epidemiology, medicine, statistics, machine learning, public health, and software engineering. The PIs have long research track records and experience leading large projects which will be required for this endeavor.

Reviewer 3
Extremely strong team with relevant expertise areas and exceptionally strong track record in NIH grant funding. Vidrine/Vidrine have overseen the development and implementation of a shared resource at a prior institution, which will serve as a model.

Investigator(s):

Weaknesses:

Reviewer 1
None noted

Reviewer 2
None.

Reviewer 3
None, team is strong with track record of success in federal funding and large grant management.

Innovation:

Strengths:

Reviewer 1
The proposal did a good job in articulating the current state of the art in tobacco cessation treatment to cancer patients throughout Florida and how the proposed AI-enhanced mHealth can help advance the current research and clinical practice paradigms by the instrumentation of mHealth as a complimentary approach to improve the efficiency in tobacco cessation treatment to cancer patients. The aim of integrating mobile health management with advanced AI and machine learning to support and enhance tobacco cessation treatment to cancer patients has the potential for broad impact to other field of research in mHealth.

Reviewer 2
The project would leverage recently developed technologies (smartphones) to offer new ways to deliver patients treatments. One-off app design for each mHealth trial is inefficient. mFLi would save development costs and make development of mHealth application possible for a wider range of researchers.

Reviewer 3
The infrastructure component of the application (to provide a one-stop-shop for app development for Florida researchers) is innovative and aims to address a major barrier in research projects. This state resource is likely to provide researchers with a competitive edge in federal grant submissions (i.e., demonstration of capacity, accelerated timelines, and ability to demonstrate milestone completion in future submissions).
The use of machine learning to analyze data is unique.

Innovation:

Weaknesses:

Reviewer 1
The broader impact of the proposed mHealth infrastructure description could benefit with illustration with a couple of exemplar technologies to be provided by the proposed mHealth infrastructure, which can help clinical researchers and practitioners to improve their efforts on the tobacco cessation treatment to cancer patients, such as some specific groups.

Reviewer 2
The proposal claims innovation for being the second study designed to develop and evaluate smoking cessation apps for cancer patients. It is not clear why focusing on cancer patients is a good idea. The patient cohort becomes much smaller when restricted to cancer patients rather than smokers generally, which can make accrual more difficult. There are existing mHealth platforms and this proposal is seeking more to add some additional functionality to these platforms (and target Florida researchers).

Reviewer 3
Minor - Development of mHealth apps themselves, including cessation apps, is not particularly innovative on its own. However, this does not detract from the infrastructure aim of the project, which could be a significant asset for researchers in the state.

Approach:

Strengths:

Reviewer 1
I enjoyed reading the proposal and the specific aims of the project are supported with well-reasoned overall strategy and methodology. The PI and his collaborators have had extensive expertise and active scholarly research accomplishments in smoking research with cancer patients, tobacco related cancer treatment and smoking cessation interventions using mobile health technologies. The application is innovative because the proposed mHealth infrastructure for Florida cancer patients (mFLi) can serve as a technology platform for researchers in divers fields of health science in Florida. Also from the proposal, it seems clear that there are no prior efforts for mHealth interventions in the area.

Reviewer 2
The investigators will test the mFLi platform with 3 demonstration projects. It is extremely important and good that they have a careful plan for testing. The testing is fairly comprehensive including assessing the ability of the apps to consent patients, collect data from wearables, randomize patients, and then analyze trial data.
The investigators have secured long term funding from Moffitt (beyond this grant duration), which will be important to ensuring that mFLi continues to be supported.

mFLi will work with both iPhones and Android, which is important for reaching a large set of patients.

Reviewer 3
Comprehensive feasibility metrics.
Randomization built into app.
Project #2 includes a comparison to the free state-wide quitline plus 10 weeks of study provided NRT, with access to data provided monthly by the quitline. This is a strong comparison condition for the RCT.
Excellent cessation trial retention rates from this group in prior cancer trials.
Strong comparator condition for RCT.

Approach:

Weaknesses:

Reviewer 1
none noted.

Reviewer 2
== Existing Tools and Open Source Licensing of Software ==
It is not clear why existing tools (such as the Insight mHealth Platform) cannot be used for the proposed smoking cessation program. This would avoid having to create mFLi from scratch and reduce costs, especially in year 1. Perhaps Insight mHealth does not contain the exact functionality that the team needs. If so, why can the team not adapt the Insight code base (or some other mobile health code base) to create mFLi?
As far as I can tell, Insight code base is not open source, which perhaps is why the team cannot build off of it (answer to my last question). Will the software for mFLi be open source? I would favor making open source licensing of the mFLi platform a requirement for funding, since otherwise a substantial amount of the infrastructure investment will not be available to researchers outside the current proposal.
If it is open source, where will the code for mFLi be released (github?). I did not see any mention of "open source" or software license for release of the code, so I am concerned this is not currently planned.

== Demonstration Project 3 ==
I have several concerns regarding "Demonstration Project 3" section. The goal of demonstration project 3 is "to identify factors associated with tobacco cessation treatment engagement, quit attempts, smoking relapses, and long-term smoking abstinence." The total sample size from Demonstration Project is n=90. The data will be divided into 70% training and 30% testing/validation. Random forests and deep learning models will be fit. "Methods based on the gini index will be used to rank the different factors' importance through permutation testing and statistical metrics will be used to assess their significance."
1) Given that the sample size is small and that the goal is to associate factors with responses (rather than achieve very high predictive performance), the use of prediction focused models (random forests and deep learning) does not seem well justified. These models are difficult to interpret and typically need a large amount of data to fit well. It would make more sense to associate potentially predictive variables with the response using univariate tests, followed by simple, interpretable multivariate models (e.g., logistic regression). In particular Demonstration project 2 states that the "study is not powered to test significant differences between our two conditions." If Demonstration project 2 with n=90 does not have sufficient power, how could demonstration project 3 have any reasonable power with only n=27 (90 x 30%) cases in the validation/test set? In fact, the situation may be even more challenging because 1/2 of the 90 patients are randomized to Quitline Treatment. For these patients many variables of interest (anything collected on the mHealth app) may not be available. Thus, the model evaluation may be based on 13.5 = 45 x 30% which is really small.
2) How will the response variables (tobacco cessation treatment engagement, quit attempts, smoking relapses, and long-term smoking abstinence) be treated (continuous, categorical, time to event, etc.) and what does this imply about modeling strategies? Several of the response variables appear to be of a time to event nature. For example, the variable "smoking abstinence" would naturally be a time to event variable with T being the time duration from treatment initiation to relapse to smoking. Successful patients (never returned to smoking) are censored at last follow

up time. This has important modeling implications because the time to event versions of random forests and deep learning which have functionality for censored observations would need to be used.
3) The proposal suggests that non-baseline variables (time dependent covariates) will be used in the predictive models "This research, which has primarily targeted non-cancer patients, has demonstrated that the consideration of a variable's slope, volatility, and magnitude of change over time can explain additional variance related to an individual's likelihood of successfully quitting, and/or early relapse." These non-baseline variables will be missing for patients who stop using the platform. The natural approach to this type of problem is to treat the response variables as time-to-event outcomes and then using survival models with time dependent covariates to predict the response event (e.g., began smoking again or stop using the platform). This is not discussed.

== Other Issues ==
1) I would prefer Demonstration Project 2 to have a larger sample size and be sufficiently powered to find a difference in treatments. Investigators hope to screen 1000 patients at each site. This seems very feasible at Moffitt (79,000 patients annually) but perhaps challenging at UF (5650 patients per year). If this is actually feasible at UF, then it feasible to screen many more patients at Moffitt in the given time frame, thus boosting overall enrollment, and pushing sample sizes up for the trial to be powered to detect efficacy.
2) Will the ML tools for Demonstration Project 3 be developed on the app and then made accessible to other users? How will the code for the models be arxived / preserved to ensure reproducibility (e.g., code posted on github)?
3) More description should be provided for the "7-day point prevalence abstinance at each follow-up" in the Demonstration Project 2 "Cessation Outcomes" section.

Reviewer 3
The required presence of research coordinators at the recruitment sites is a significant limitation and cost burden for long term use. An automated process at patient check-in (folded into 'typical' patient check-in procedures) would be more sustainable.
Currently seems worded to capture traditional smokers (i.e., criteria is "report smoking 1+ cigarette in past 30 days) and ignores other types of nicotine users (including possibly dual users based on the sample treatment messaging).
The sequencing of the demonstration projects is a bit unclear. If goals are not met for Demonstration project #1 (e.g., ≥90% screening completion, ≥30% enrolled in treatment), will Project #2 start? If goals of Project #1 are not met, will adjustments be made and impact re-evaluated?
In Project #2, it is unclear why participants need a valid home address to be eligible and this would seem to exclude many disadvantaged individuals who experience high smoking rates.
Content delivery is video-based rather than allowing the viewer to select their preferred delivery method (e.g., select text, video).
Some level of iterative design with feedback from end users might strengthen the approach.
What happens if Table 3 benchmarks are unmet?
Lack of biochemical verification of cessation in the RTC is a major limitation, and if the app is designed for smoking cessation trials, then a method of verification of smoking status seems fundamental to the app design

---

<u>Environment:</u>

Strengths:

Reviewer 1
The team has strong institutional support and resources to develop the proposed mFLi infrastructure, which consists of a software platform to create mHealth tools, including Web-based and native apps. The proposed mFLi will provide development environment, testing environment and production environment and allow users to create, test and publish apps, in addition to provide access to a mobile developer and UI/UX designer.
To demonstrate the usability and deployment of the mFLi infrastructure, the proposed project will develop three pilot projects:
(1) to efficiently testing mHealth strategies to assess and recruit cancer patients who smoke
(2) to efficiently connect the cancer patients who smoke with existing and newly developed Digital intervention programs.

(3) to offer machine learning methods and tools to analyze data collected through the mFLi infrastructure.

Reviewer 2
The research will take place at Moffitt, Miami, and UF. These institutions have deep experience with clinical trials and mobile health. By enrolling patients at several sites 1) the research team will gain experience deploying the mHealth application in a multi-center trial environment which is important prior to using the platform on a larger scale and 2) reach larger and more diverse patient populations.

Reviewer 3
Existing center for digital health at Moffitt will lend support to the mFLi development. Institution has necessary IT support and staffing via software developer, ux/ui developer, program manager, and IT support. Institutional commitment to sustainability.

Environment:

Weaknesses:

Reviewer 1
The third project on developing machine learn methods to help users of the proposed mHeath infrastructure can benefit from more elaboration. Compared to the other two projects, the third one is weaker and the proposed example analytic approach is mainly random forest, which is conventional method.

Reviewer 2
None.

Reviewer 3
None

Health Impact:

Strengths:

Reviewer 1
The proposed mHealth infrastructure (mFLi) will contribute to Florida from two broader aspects: (1) It will advance the technology and clinical practice for tobacco cessation treatment to cancer patients in Florida, and (2) it will have the potential to facilitate the public health in Florida. For example, the proposed mHealth research infrastructure will have a long-term impact on advancing the methods and technology capability of integrating advanced mobile computing and machine learning for delivering the next generation of mHealth in Florida.
The proposal outlines the security, privacy and compliance plan, including HiPPA compliance, NIST 800 standards, with respect to the credentialed users. Data will be encrypted at both file and platform level.

Reviewer 2
A successful project will clearly have a positive impact on health. This positive impact will come in two forms: development of mFLi which will facilitate more mHealth interventions / trial in Florida and evidence to support using the mHealth app for smoking cessation (over Quitline).

Reviewer 3
Focus is on increasing access to treatment and removing barriers to treatment. Directly relevant to tobacco related disease and treatment with potential for broader applications in the future

---

Health Impact:

Weaknesses:

Reviewer 1
The proposed infrastructure will be evaluated through the development and evaluation of each of the three pilot projects. The proposal could benefit more by elaborating on the overall evaluation of the mFLi by cross-evaluation of all three projects.

Reviewer 2
Estimates of the mortality/morbidity impact that statewide use of mHealth (instead of Quitline) would strengthen the proposal. For example, suppose Quitline has an estimated cessation effectiveness of 20%. Perhaps mHealth could raise this to 30%. How much would a 10% increase in effectiveness matter in terms of extra patient years of life?

Reviewer 3
None.

OVERALL IMPACT

Strengths:

Reviewer 1
The proposed mHealth infrastructure development, including the software, the testing and the production environments and the three pilot projects, will have broader impact not only on supporting and improving the efficiency of tobacco cessation treatment to cancer patients but also the mHealth interventions in general in Florida. The proposed mFLi infrastructure will advance science and technology in the tobacco cessation treatment of cancer patients and also improve clinical practice of smoking cessation efficiency in Florida through integrating advanced mHealth with advances in machine learning.

Reviewer 2
The strengths are detailed in each impact response.

Reviewer 3
The proposal aims to enhance access mHealth infrastructure for Floridian researchers. The goal is to provide an easily customizable app framework, and second to have that app function for recruitment and screening into research trials. Highly significant to patients and researchers. Highly likely to increase competitiveness in grants submissions

---

OVERALL IMPACT

Weaknesses:

Reviewer 1
None noted.

Reviewer 2
The main issues to be addressed are: 1) What license will mFLi be released under? Will this be open-source? 2) Several deficiencies noted in Machine Learning modeling proposed in Development Project 3.

Reviewer 3
None, will be an impressive and impactful infrastructure resource.

Protections for Human Subjects:

Description of any concerns and recommendations:

Reviewer 1
None noted

Reviewer 2
The proposal involves human subjects who will be randomized to one of two tobacco cessation programs. The overall risks to patient health seems low. The clinical trial portion of the proposal does not propose early stopping for safety or efficacy. This seems appropriate given the relatively small sample size (n=90) and the low risk nature of the intervention. The main risk to subjects would be unintentional release of PHI. The proposal minimizes this risk with the proposed mHealth technology.

Reviewer 3
No concerns.

Vertebrate Animals:

Description of any concerns and recommendations:

Reviewer 2
The proposal will not study animals.

Budget and Period of Support:

Recommended modifications (if any):

Reviewer 1
None noted.

Reviewer 2
None.

Reviewer 3
No concerns.

Scientific or Budget Overlap:

Description of any concerns and recommendations for follow-up and/or modifications:
Reviewer 1
None noted.

Reviewer 2
I do not see any problem with the budget or overlap with other projects held by the investigators.

Reviewer 3
No concerns.

ADDITIONAL RECOMMENDATIONS OR OTHER COMMENTS

Beyond the items already addressed, is there any additional advice you are willing to offer the applicant?

There are no Comments for this question.

ADDITIONAL REVIEW CONSIDERATIONS - Separately from the overall impact score, peer reviewers will rate the following items.

Tobacco Relatedness:

Strengths:

Reviewer 1
The mFLi infrastructure is developed for improving and supporting efficient delivery of the tobacco cessation treatment to cancer patients in Florida, a high relevance to Tobacco relatedness. The team has extensive expertise in mHealth smoking cessation interventions to special populations, including underrepresented groups, and machine learning in oncology.

Reviewer 2
The proposal proposes interventions for tobacco cessation in patients who have cancer. Successful cessation programs will reduce use of tobacco among cancer patients and likely reduce mortality and morbidity.

Reviewer 3
Tobacco cessation is directly relevant to general health concerns and cancer specifically, and mHealth has the potential to reach a broader range of patients in need of support for cessation.

Tobacco Relatedness:

Weaknesses:

Reviewer 1
None noted.

Reviewer 2
None

Reviewer 3
None.

Infrastructure (if applicable):

Strengths:

Reviewer 1
The proposed mFLi infrastructure will be made available to researchers throughout Florida. The project has laid out a good plan for a scientific advisory committee and a community advisory committee as required.

Reviewer 2
The proposal will enable researchers to develop their own apps in low code / no code inferface, using the mFLi platform. The proposal includes a Scientific Advisory Board (SAB) whose members have a diverse set of skill sets including ones highly relevant to the proposed application such as the Senior Vice President and Chief Digital Officer

at Moffitt. The proposal includes plans to create a Community Advisory Board at each site (Moffitt, UF, Miami). Additional funding provided by Moffitt will ensure that mFLi is supported beyond the life of this grant.

Reviewer 3
Will provide excellent pilot and feasibility data for future grant submissions.
Connects three large hospital systems with large patient numbers.

Infrastructure (if applicable):

Weaknesses:

Reviewer 1
None noted.

Reviewer 2
There does not appear to be any plan to release the code used for creating the mFLi platform. If this code is not released with an open source license in an easily accessible well-documented format, then a substantial amount of the infrastructure value will not be available to researchers who would like to extend / modify the mFLi platform to enable creation of apps with different / new features not supported in the current version of mHealth.

Reviewer 3
None.
